# Maternal Nutrition during Early Pregnancy and Cardiometabolic Status of Neonates at Birth

**DOI:** 10.1155/2018/7382946

**Published:** 2018-04-24

**Authors:** Musarrat Riaz, Fareeha Shaikh, Asher Fawwad, Rubina Hakeem, A. Samad Shera, Graham A. Hitman, Bishwajit Bhowmik, Nayla Christina do Vale Moreira, Abdul Basit, Akhtar Hussain

**Affiliations:** ^1^Department of Medicine, Baqai Institute of Diabetology and Endocrinology, Baqai Medical University, Karachi, Pakistan; ^2^GIFTS Project, Karachi, Pakistan; ^3^Department of Community Medicine, Institute of Health and Society, University of Oslo, Oslo, Norway; ^4^Department of Biochemistry, Baqai Medical University, Karachi, Pakistan; ^5^Department of Research, Baqai Institute of Diabetology and Endocrinology, Baqai Medical University, Karachi, Pakistan; ^6^Department of Education, Government of Sindh, Karachi, Pakistan; ^7^Diabetic Association of Pakistan, WHO Collaborating Centre, Karachi, Pakistan; ^8^Blizard Institute, Barts and The London School of Medicine and Dentistry Queen Mary University of London, London, UK; ^9^Centre for Global Health Research, Diabetes Association of Bangladesh, Dhaka, Bangladesh

## Abstract

**Objective:**

To explore the impact of maternal body weight on maternal nutrition and micronutrient status in early pregnancy and potential impact on metabolic status in newborns.

**Methodology:**

The EU FP7 project GIFTS was conducted from Jan 2012 to May 2014. Demographic details and anthropometric measurements of women in the first trimester of pregnancy were obtained. Blood samples were collected for OGTT, insulin, lipid profile, serum folate, ferritin, vitamin D, vitamin B12, and red cell folate. Newborn anthropometric characteristics were observed. Cord blood samples were collected after delivery for glucose, insulin, and lipid profile of newborns.

**Results:**

A total of 301 pregnant mothers, 108 overweight, 63 underweight, and 130 normal weight were included. Prevalence of vitamin D deficiency (<30 ng/mL) and low vitamin B12 (<190 ng/l) were high, 44% and 42%, respectively, in the first trimester. Anemic women (due to B12 or iron deficiency) were 79%, while 72% had low ferritin levels. Gestational diabetes was 16%. Differences were observed between underweight and overweight mothers (*P* < 0.05) for fasting blood glucose, insulin levels, and serum ferritin were observed. No significant difference was observed between vitamin D, serum B12, and red cell folate levels.

**Conclusion:**

Prevalence of multiple micronutrient deficiencies was common among Pakistani women during early pregnancy despite the nonvegetarian diet that has important implications for pregnancy care in Pakistan and potentially in expatriate communities living abroad. This trial is registered with ISRCTN number 83599025.

## 1. Introduction

Noncommunicable diseases (NCDs) including diabetes and cardiovascular diseases (CVD) are the leading causes of morbidity, disability, and mortality worldwide [1, 2]. Evidence from observational, experimental, and epidemiological studies suggests a link between maternal nutrition during pregnancy and susceptibility to chronic diseases in adult life [2, 3]. Suboptimal fetal growth has been linked to a higher risk of cardiovascular diseases (CVD), hypertension, insulin resistance, and type 2 diabetes in several populations [4, 5].

Association between an adverse intrauterine environment (initially using low birth weight as its manifestation) and an increased risk of type 2 diabetes, impaired glucose tolerance, and coronary heart disease later in life was first observed in the 1980s and has been widely reported since, in theories of “fetal programming” [6–8], maternal undernutrition, and specific micronutrient deficiencies related to CVD, insulin resistance, and type 2 diabetes. More recently, studies have reported that states of “overnutrition,” represented by diabetes in pregnancy, and/or overweight/obesity during pregnancy, may also predispose offspring to a higher incidence of chronic diseases [9–12].

The mechanisms by which nutrition perturbations contribute to intrauterine fetal programming are poorly understood, but may have lifelong consequences. It is hypothesized that alteration in fetal nutrition and endocrine status may result in adaptation in physiologic and metabolic status thereby predisposing the individual to future metabolic endocrine and cardiovascular diseases in adult life [13]. Although the importance of maternal nutrition to fetal development and birth outcomes has been demonstrated in experimental animal studies, the findings of such studies in humans are much less consistent, due to many confounding factors within and between studies, including varied baseline maternal nutritional status and behavior (e.g., food and dietary practices), genetic factors, socioeconomic status (SES) of the study population, timing, and methods of assessing or manipulating maternal nutritional variables.

The main focus of most of the studies has been on perinatal undernutrition and specific nutrient deficiencies [14]. Studies have shown that for a given BMI, South Asians have greater fat mass termed as thin-fat insulin-resistant phenotype, which may be partly responsible for increasing the prevalence of type 2 diabetes [15, 16]. Recent evidences suggest that this phenotype is possibly present at birth [17]. Studies done in India and other South East Asian countries may not be applicable to Pakistan as dietary patterns vary from country to country specially in regard to vegetarian and nonvegetarian components [18]. Data from Pakistan in this regard is scarce. Hence, the present study was planned to explore the impact of maternal nutrition and micronutrient status in early pregnancy on metabolic status in newborns at birth.

## 2. Patients and Methods

This is a substudy of a prospective multicenter EU FP7 project GIFTS (genomic and lifestyle predictors of fetal outcome relevant to diabetes and obesity and their relevance to prevention strategies in South Asian peoples) project conducted from January 2012 to May 2014 in Karachi, Pakistan. Similar studies were also carried out in Bangladesh and London. In Pakistan, patients were recruited from 6 randomly selected antenatal clinics including both primary and secondary care hospitals of Karachi, Pakistan. Pregnant subjects who are in the first trimester of their pregnancy (≤13-week gestation), singleton, conceiving without treatment of fertility and were willing to participate in the study were requested and included in the study after their written consent. Pregnant subjects at inclusion with type 2 diabetes, history of gestational diabetes, or pregnancy-induced hypertension were not included in the study.

Duration of gestation was calculated from the date of last menstrual period and ultrasonographic examination at the time of recruitment. In case of disparity, ultrasonographic estimation was used. Each study participant had 3 visits during the study period: 1st visit in the first trimester at the time of registration between 10 and 14 weeks, 2nd visit between 24 and 28 weeks of gestation, and 3rd visit at delivery.

Maternal demographic details, medical history, pregnancy history, and anthropometric measurements were obtained from the mother at the baseline visit using a structured questionnaire and interview. Participant socioeconomic status was assessed by household monthly income and educational level, and employment status was also registered. Data regarding age, parity, history of diabetes, and hypertension were obtained by interview.

Maternal height, weight, and midarm circumference (MAC), of the left upper arm, measured at the midpoint between the tip of the shoulder and the tip of the elbow, were recorded. Bicep and tricep skinfold thickness, on the midline of the anterior (front) surface of the arm (over the bicep muscle) and on the midline of the posterior (back) surface of the arm (over the tricep muscle), were measured, respectively. A vertical pinch, parallel to the long axis of the arm, made at the landmark, was recorded at the time of registration. Maternal height was obtained at the first visit, and weight was measured by a calibrated scale at each visit. Maternal BMI (kg/m^2^) was then calculated in the subsequent measures based on the appraisal of the first measures of the maternal height [19].

Fasting blood samples were collected at both visits (1st and 2nd) for glucose (fasting and 2-hour post 75 gm glucose load), insulin, lipid profile, and circulating micronutrients including serum folate, ferritin, vitamin D (25-hydroxyvitamin D) and vitamin B12, and red cell folate. Plasma glucose concentration was measured by glucose oxidase method. Serum insulin, leptin, ferritin, folate, and vitamin B12 concentrations were analyzed by ELISA/chemiluminescent immunoassay. The vitamin D status was determined by ELISA method while red cell folate (RBC folate) was measured by ELISA/chemiluminescent immunoassay. Total cholesterol was estimated by CHOD-PAP method whereas triglycerides were measured by GOP_PAP method. Low- and high-density lipoprotein values were estimated by homogeneous enzymatic colorimetric method. Hemoglobin values were collected from routine clinical testing documented in patient's hospital records. The inter- and intra-assay coefficients of variability for glucose (8.8% and 4.5%), cholesterol (9.2% and 4.4%), triglycerides (7.3% and 5.2), HDL (6.4% and 3.9%), LDL (5.5% and 3.3%), serum folate (5.2% and 2.3%), ferritin (5.8% and 2.9%), vitamin D (6.6% and 4.2%), vitamin B12 (5.1% and 3.9%), and RBC folate (4.9% and 2.2%) were in acceptable ranges.

Maternal glycemic status was assessed using self-reported history of diabetes that preceded the pregnancy. An OGTT with a 75 gm oral glucose tolerance test (OGTT) was carried out at inclusion in the 1st trimester and in the second trimester (24–28 weeks). Gestational diabetes was diagnosed if the woman has either a fasting plasma glucose level of 100 mg/dL or above or a 2-hour plasma glucose level of 140 mg/dL or above [20]. Weight gain during pregnancy was derived from the difference between maternal weight at first visit and the last visit weight from the chart, prior to delivery. Maternal nutritional intake was evaluated on the first visit utilizing a validated semiquantitative food frequency questionnaire (FFQ). FFQ data was analyzed to estimate average daily intake as per standard norms (e.g., following data extraction and cleaning, frequencies were standardized to “per day” and multiplied by standard serving sizes (grams)) [21]. Micronutrient assessment included serum ferritin, serum folic acid, serum B12, red cell folate, and vitamin D levels.

Detailed information about the study was given to the participants, and informed consent was obtained following the Helsinki declaration and standard research and data governance procedures. The ethical approval of the study was obtained from the Institutional Review Board (IRB) of Baqai Institute of Diabetology and Endocrinology (Ref. 12/31/09/0460).

### 2.1. Newborn Characteristics

Newborn characteristics including sex, gestational age at birth, and the placental weight were recorded at the time of delivery. Newborn outcomes of interest included anthropometric characteristics (length, birth weight, head circumference, abdominal circumference, chest circumference, mid-upper arm circumference (MUAC), and subscapular bicep and tricep skinfold thickness). Newborn length was measured on a length board. Tricep skinfold measurement was taken from the midpoint between the acromion (shoulder) and olecranon processes (elbow) on the posterior surface of the left upper arm by holding a vertical fold of skin and subcutaneous tissue with the help of the thumb and fingers. Subscapular skinfold measurement was taken from the inferior angle of the scapula by holding a diagonal fold of skin and subcutaneous tissue below and medial to the inferior angle of the scapula. All skinfolds were taken on the left side of the body using digital fat track II skinfold caliper. The head, mid-upper arm, chest, and abdomen circumferences were measured using a nonstretchable tape. Percent body fat was derived from measures of tricep and subscapular skinfold thickness [22]. Anthropometric measurements of the newborns were collected within 24 hours of the birth.

Samples of cord blood of newborns were collected immediately after delivery, and cord serum analyzed for glucose, serum insulin, lipid profile, and glucose-to-insulin ratio was calculated. Cord blood serum was also analyzed for vitamin B 12, serum ferritin, serum folate, RBC folate, and vitamin D levels. Methods used for the analysis of cord blood were the same as those used for the analysis of maternal blood (described above).

### 2.2. Statistical Analysis

Data was presented as the mean ± standard deviation (SD) or in terms of frequency with percentage (%). Data was analyzed using Statistical Package for Social Sciences (SPSS) version 17.0. ANOVA or *t*-test was used for the comparison between continuous variables while chi-square test was used for categorical variables. Binary logistics regression was used for predicting crude birth weight of neonates.

## 3. Results

A total of 301 pregnant mothers were included in the study. Mean maternal age was 24.78 ± 4.89 years with the mean maternal BMI of 22.45 ± 4.59 kg/m^2^. There were 108 overweight (35.9%), 63 underweight (20.9%), and 130 normal weight (43.2%) women in the study. Baseline gestational age was 9.78 ± 2.29 weeks.

The demographic details of the subjects are presented in Table 1. Mean gestational age at delivery was 38.39 ± 2.13 weeks. Gestational diabetes was diagnosed in 47 (15.9%) subjects with the highest prevalence in overweight mothers. Underweight mothers were younger than the mothers with normal weight. Micronutrient deficiency was common among recruited mothers in early pregnancy. The deficiency level for 25-OH vitamin D < 30 ng/mL was found in 44%, and deficiency for B12 was recorded in 42% of the mothers, while anemia was present in 79%. Low ferritin existed in 72% of the recruited mothers.

A significant difference (lower values) in fasting blood glucose and insulin levels was noted between underweight and overweight mothers (*P* < 0.05). Triglyceride, cholesterol, serum ferritin, LDL, and homocysteine levels were lower in underweight compared to normal weight and overweight mothers. No significant difference was observed between vitamin D, serum B12, and red cell folate levels among these groups (Table 2).

Baseline characteristics of the newborns are shown in Table 3. All newborns were delivered at full term, with a mean weight of 2.85 ± 0.50 kg. LBW (low birth weight defined as <2.5 kg) infants were shorter 43.57 ± 3.50 cm versus 46.81 ± 3.14 cm (*P* < 0.05) and lighter 2.01 ± 0.29 versus 3.00 ± 0.36 kg (*P* < 0.05). Of 121 neonates, the frequency of low birth weight was 18 (14.8%). Abdominal and chest circumference measurements were significantly lower in LBW babies (*P* < 0.05) (Table 3); all other anthropometric measurements were not significant. Cord blood samples were collected immediately after delivery. Micronutrient deficiency was highly common in neonates. Deficiency levels of vitamin D (<30 ng/ml) were in 77.4% and B12 (<190 pg/ml) in 74.7% while serum ferritin (<11 ng/ml) was present in 4.8% at delivery.

Amongst the underweight infants, there was a trend for a lower blood glucose levels than neonates born with normal weight (71.5 ± 23.38 mg/dl versus 75.8 ± 38.58 mg/dl) but the difference was insignificant. LBW infants also had a nonsignificant lower plasma insulin levels (5.62 ± 7.74 versus 29.05 ± 67.82 mmol/l, Table 4). Similarly, triglyceride levels were also found to be lower in LBW infants (56.4 ± 58.68 mg/dl) compared to NBW infants (62.54 ± 72.5 mg/dl). Moreover, glucose-to-insulin ratio was lower in LBW newborns when compared with NBW infants (33.91 ± 45.17 versus 37.67 ± 62.67; *P* > 0.01), and homeostasis model assessment of insulin resistance (HOMA-IR) suggests that LBW infants were more insulin sensitive than NBW infants. Vitamin B12 levels were lower in LBW compared to NBW infants (192.6 ± 143.51 versus 241.42 ± 215.01 pg/ml; *P* value > 0.05). Also, serum ferritin levels and serum folate levels were also low in LBW babies (156.36 ± 135.46 versus 199.09 ± 233.77 ng/ml and 15.51 ± 7.04 versus 17.47 ± 7.67 ng/ml; *P* value > 0.05), respectively. There was no significant difference between vitamin D, renin, leptin, and homocysteine levels amongst the LBW and NBW babies (Table 4).

Three different binary logistic regression models were used for the prediction of crude birth weight of neonates. The first model included mother's age, gestational age (at baseline), parity, BMI, FBS, RBS, TG, vitamin B12, and vitamin D. None of them showed significance in predicting neonate's birth weight. Similar results were obtained in other two models in which parity, BMI, FBS, RBS, and triglycerides were eliminated for the second model, and all variables were eliminated except for vitamin B12 and vitamin D for the third model (Table 5). GDM cases were excluded from this cohort and therefore not included in the regression model.

No significant difference was observed for a mother's biochemical data according to birth weight category of neonates for triglycerides, vitamin B12 and vitamin D levels, FBS, RBS, HOMA-IR, and serum insulin.

## 4. Discussion

Our study reports the frequency of micronutrient deficiencies and metabolic status during early pregnancy and its consequences on newborns at birth with special reference to birth weight and cardiometabolic status.

Adverse consequences of LBW have been recognized for a long time especially for acute complications [23]. Our findings indicate that glucose metabolism in LBW newborn is modified with significantly lower plasma glucose and insulin levels than normal birth weight babies. This occurs in the absence of any perinatal complications. This observation is in accordance with a number of previous studies reporting that glucose metabolism and insulin action are profoundly modified in low birth weight situation [4]. Economides et al. [24] measured plasma glucose and insulin in the third trimester SGA and AGA fetuses by cordocentesis. They found lower glucose and insulin, as well as higher G/I ratio, in growth-retarded fetuses. We observed similar findings in our study population. Additional factors related with insulin production by fetus may explain the findings of our study in regard to LBW newborns. However, considering the low number of babies in our cohort, further large-scale studies are needed with appropriate sample size.

We observed that the prevalence of multiple micronutrient deficiencies was common among Pakistani women during early pregnancy. In our study, micronutrient status of pregnant mothers was assessed during the first trimester of pregnancy (G. age 9.78 ± 2.29 wks.); therefore, the chances of confounding effect of hemodilution which is common in late pregnancy were unlikely.

Serum ferritin levels are primarily a reflection of iron status of the mothers. In this study, we observed that around 72% of pregnant women had deficient concentration of ferritin in the blood, reflecting depleted iron stores in the body during early pregnancy. Previous studies have reported an association between depleted iron stores in mothers during early pregnancy and anthropometric measurements including weight, length, and midarm circumference of the newborn at birth [25]. These findings need to be explored in future longitudinal studies. Moreover, anemia was present in nearly 79% of the study population. Similar results were reported in other studies of the region. For instance, in China, prevalence of anemia during pregnancy was found in 80% of the women. Likewise, an Indian study also demonstrated high prevalence of anemia in their study population.

For a nonvegetarian population, there were unexpectedly high levels of vitamin B12 deficiency compared to the previously reported figures of 17% (back up with references). We observed that nearly 47% of the women were deficient in vitamin B12 when they started pregnancy. Studies in Pune, India, have demonstrated a higher prevalence of vitamin B12 deficiency during early pregnancy [26, 27], but this is likely to be attributable to the vegetarian (including lacto-vegetarian) diet in this Indian population [28, 29]. Despite the nonvegetarian diet, high level of B12 deficiency in this population may have existed due to lower consumption of fish in Pakistani traditional food. In the offspring, the lowest B12 levels were found in those of LBW.

Folate is an integral component of cell proliferation and DNA synthesis. Studies have shown that folate deficiency during pregnancy may have adverse effect in rapidly dividing cells of the developing conceptus [30] and is associated with an increased risk of congenital anomalies [31]. In our study, more than fifty percent of the mothers were deficient in serum folate and approximately seventy percent had deficiency of red cell folate in the blood.

Our results reported that 44.5% of the mothers had deficiency of vitamin D (range of 25-OH vitamin D). However, almost the same percentage (44.9%) was found in the category of insufficiency of circulating vitamin D. Unlike our result, another Pakistani study has shown high prevalence of vitamin D deficiency in women during pregnancy. The difference in the results might be because they assessed vitamin D levels in the 3rd trimester whereas we have analyzed vitamin D levels in early pregnancy. Furthermore, difference in the unit of assessment and cutoff values to describe deficiencies may be another possible reason for this variation in the results [32].

We could not detect a significant difference in levels of serum ferritin, vitamin B12, and serum folate in LBW babies compared to NBW. This may have owing to low statistical power. Maternal under nutrition during pregnancy lead to diminished placental and fetal growth in both animal and humans [2, 3, 33]. Fetal growth is vulnerable to both early pregnancy and maternal dietary intake and micronutrients during the period of rapid placental development [18, 27–29]. The mean triglycerides and the HDL levels were low in all the participants, and triglyceride level was significantly different between all the three groups based on BMI. Besides adverse pregnancy outcomes, maternal dyslipidemia during pregnancy may also have long-term consequences for offspring's cardiovascular health. Early pregnancies characterized by lower triglyceride (TG) levels more often resulted in babies with low birth weight while dyslipidemia in pregnancy was associated with adiposity at birth, both of which are outcomes associated with atherosclerotic disease later in life [34, 35].

## 5. Conclusion

High prevalence of micronutrient deficiency was found in pregnant women in their early pregnancy and also amongst the neonates irrespective of their birth weight or mothers' BMI. Therefore, nutritional assessment in early and prepregnancy is vital, and necessary supplementation is recommended. The data presented here has important implications for pregnancy care in Pakistan and potentially in expatriate communities living abroad.

## Figures and Tables

**Table 1:** Baseline characteristics at first visit and anthropometric measurements of mothers.

Variables	Overall	Underweight (BMI ≤ 18.5)	Normal (BMI 18.6–23)	Overweight (BMI ≥ 23)
*n*	301	63	130	108
Age (years)^†♦^	24.78 ± 4.89	23.52 ± 5.08	24.34 ± 4.68	26.05 ± 4.79
Gestational age (weeks)				
At first visit	9.78 ± 2.30	9.54 ± 2.40	9.79 ± 2.25	9.92 ± 2.30
At delivery^†♦^	38.39 ± 2.13	37.97 ± 2.18	38.09 ± 1.8	38.98 ± 2.37
Gestational diabetes^∗^^†♦^	47 (15.9%)	6 (9.7%)	16 (12.6%)	25 (23.4%)
Parity				
Primary	85 (29.1%)	24 (40.0%)	35 (27.8%)	26 (24.5%)
Multi	207 (70.9%)	36 (60.0%)	91 (72.2%)	80 (75.5%)
Weight (kg)^∗^^†♦^	54.37 ± 11.87	42.31 ± 4.57	50.53 ± 4.78	66.02 ± 10.71
Height (cm)	155.95 ± 5.82	156.69 ± 6.04	155.65 ± 5.57	155.87 ± 6.00
BMI (kg/m^2^)^∗^^†♦^	22.45 ± 4.69	17.23 ± 1.07	20.95 ± 1.34	27.31 ± 4.02
Waist circumference (cm)^∗^^†♦^	74.13 ± 9.63	64.06 ± 4.13	71.67 ± 5.44	82.97 ± 8.27
Hip circumference (cm)^∗^^†♦^	91.48 ± 10.57	81.02 ± 5.16	88.78 ± 7.02	100.82 ± 8.79
WHR^∗^^†♦^	0.81 ± 0.05	0.79 ± 0.04	0.81 ± 0.04	0.82 ± 0.05
MUAC (cm)^∗^^†♦^	25.07 ± 3.56	21.77 ± 1.98	24.19 ± 2.41	28.09 ± 3.14
Bicep thickness (mm)^†♦^	13.35 ± 12.14	11.39 ± 13.45	11.49 ± 9.55	18.37 ± 13.69
Tricep thickness (mm)^∗^^†♦^	19.96 ± 12.65	13.48 ± 10.84	20.16 ± 12.72	27.02 ± 10.67
Systolic BP (mmHg)^†♦^	107.48 ± 9.33	104.84 ± 9.63	105.65 ± 9.26	111.21 ± 8.03
Diastolic BP (mmHg)^†♦^	70.61 ± 7.31	69.44 ± 6.10	70.00 ± 7.39	72.04 ± 7.67

Data presented as the mean ± SD or *n* (%); *P* value < 0.05 is considered statistically significant; ^∗^significance difference b/w underweight and normal weight; ^†^significance difference b/w underweight and overweight; ^♦^significance difference b/w normal weight and overweight.

**Table 2:** Baseline biochemical and micronutrients of mothers at first visit.

Variables	Overall	Underweight (BMI ≤ 18.5)	Normal (BMI 18.6–23)	Overweight (BMI ≥ 23)
*n*	301	63	130	108
Fasting OGTT (mg/dl)^†^	84.54 ± 11.57	82.30 ± 10.54	84.41 ± 12.54	86.00 ± 10.79
2-hour post OGTT (mg/dl)^†♦^	113.14 ± 28.03	105.29 ± 24.14	108.90 ± 24.16	122.72 ± 31.72
Cholesterol (mg/dl)^∗^^†♦^	165.06 ± 35.03	153.25 ± 32.37	164.16 ± 35.42	173.04 ± 34.80
Triglycerides (mg/dl)^∗^^†♦^	74.03 ± 32.27	60.68 ± 21.86	71.51 ± 29.02	84.87 ± 37.38
HDL (mg/dl)^∗^	46.07 ± 12.04	43.73 ± 10.07	48.04 ± 14.25	45.07 ± 9.69
LDL (mg/dl)^†♦^	96.05 ± 29.73	91.58 ± 30.10	93.64 ± 27.48	101.57 ± 29.27
Serum insulin (*μ*IU/ml)^†^	9.06 ± 5.46	7.61 ± 4.05	9.10 ± 5.57	9.87 ± 5.89
HOMA-IR^†^	1.91 ± 1.23	1.55 ± 0.89	1.91 ± 1.28	2.12 ± 1.29
Vitamin B12 (pg/ml)	249.88 ± 188.86	280.52 ± 200.14	249.49 ± 199.12	232.48 ± 167.74
Deficiency (<190 pg/ml)	126 (41.9%)	23 (36.5%)	55 (42.3%)	48 (44.4%)
Serum ferritin (ng/ml)	15.00 ± 20.53	11.84 ± 15.24	14.51 ± 21.49	17.45 ± 21.88
Deficiency (<11 ng/ml)	165 (54.8%)	43 (68.3%)	76 (58.5%)	46 (42.6%)
Serum folate (ng/ml)	4.98 ± 4.50	5.52 ± 5.21	4.80 ± 4.46	4.87 ± 4.11
Deficiency (<2 ng/ml)	20 (6.6%)	4 (6.3%)	6 (4.6%)	10 (9.3%)
Vitamin D (ng/ml)	16.35 ± 12.91	17.48 ± 12.27	15.30 ± 11.76	16.97 ± 14.53
Deficiency (<30 ng/ml)	266 (88.4%)	55 (87.3%)	117 (90.0%)	94 (87.0%)
RBC folate (ng/ml)	164.97 ± 253.62	152.89 ± 218.9	192.09 ± 302.68	139.36 ± 201.21
Deficiency (<140 ng/ml)	216 (71.8%)	46 (73.0%)	89 (68.5%)	81 (75.0%)

Data presented as the mean ± SD or *n* (%); *P* value < 0.05 is considered statistically significant; ^∗^significance difference b/w underweight and normal weight; ^†^significance difference b/w underweight and overweight; ^♦^significance difference b/w normal weight and overweight.

**Table 3:** Baseline characteristics of neonates.

Variables	Overall	Low birth weight (weight < 2.5 kg)	Normal birth weight (weight ≥ 2.5 kg)
*n*	121	18 (14.8%)	103 (85.2%)
Weight (kg)^∗^	2.85 ± 0.50	2.01 ± 0.29	3.00 ± 0.36
Gender			
Male	62 (53.9%)	7 (41.2%)	55 (56.1%)
Female	53 (46.1%)	10 (58.8%)	43 (43.9%)
Length (cm)^∗^	46.34 ± 3.37	43.57 ± 3.50	46.81 ± 3.14
Head circumference (cm)^∗^	33.98 ± 1.57	32.54 ± 1.76	34.23 ± 1.41
MUAC (cm)^∗^	10.81 ± 1.24	9.43 ± 0.76	11.05 ± 1.15
Abdominal circumfernce (cm)^∗^	32.14 ± 2.25	30.25 ± 2.42	32.46 ± 2.07
Chest circumference (cm)^∗^	33.05 ± 1.76	31.64 ± 1.68	33.29 ± 1.67
Bicep (mm)	3.15 ± 2.03	2.86 ± 2.24	3.21 ± 2.01
Tricep (mm)	3.39 ± 1.75	2.75 ± 1.19	3.51 ± 1.81

Data presented as the mean ± SD or *n* (%); *P* value < 0.05 is considered statistically significant; ∗ sign denotes significant difference.

**Table 4:** Baseline biochemical and micronutrients of neonates (cord blood sample).

Variables	Overall	Low birth weight (weight < 2.5 kg)	Normal birth weight (weight ≥ 2.5 kg)
*n*	121	18	103
Serum glucose (mg/dl)	75.27 ± 36.96	71.5 ± 23.38	75.8 ± 38.58
Cholesterol (mg/dl)	81.36 ± 52.93	57.4 ± 13.96	84.59 ± 55.43
Triglycerides (mg/dl)	61.81 ± 70.71	56.4 ± 58.68	62.54 ± 72.5
HDL (mg/dl)	24.9 ± 10.19	19.1 ± 6.72	25.69 ± 10.36
LDL (mg/dl)	42.81 ± 35.3	28.4 ± 10.15	44.76 ± 37.03
Serum insulin (*μ*IU/L)	26.26 ± 64.11	5.62 ± 7.74	29.05 ± 67.82
HOMA-IR	3.03 ± 7.11	1.31 ± 2.27	3.27 ± 7.53
Homocysteine (*μ*mol/L)	8.38 ± 14.61	4.11 ± 3.04	9.02 ± 15.54
Surplus (≥15 *μ*mol/L)	6 (50.6%)	0 (0%)	6 (49.3%)
Serum renin (pg/ml)	177.89 ± 150.79	133.46 ± 90.62	183.97 ± 156.71
Deficiency (<83.35 pg/ml)	22 (26.5%)	3 (30.0%)	19 (26.0%)
Serum leptin (ng/ml)	11.75 ± 11.27	9.48 ± 13.18	12.08 ± 11.04
Deficiency (<4.8 ng/ml)	19 (23.8%)	6 (60.0%)	13 (18.6%)
Vitamin B12 (pg/ml)	235.54 ± 207.63	192.6 ± 143.51	241.42 ± 215.01
Deficiency (<190 pg/ml)	62 (74.7%)	9 (90.0%)	53 (72.6%)
Serum feritin (ng/ml)	194.01 ± 224.16	156.36 ± 135.46	199.09 ± 233.77
Deficiency (<11 ng/ml)	4 (4.8%)	2 (20.0%)	2 (2.7%)
Serum folate (ng/ml)	17.23 ± 7.58	15.51 ± 7.04	17.47 ± 7.67
Deficiency (<2 ng/ml)	0 (0%)	0 (0%)	0 (0%)
Vitamin D (ng/ml)	18.91 ± 14.33	19.12 ± 11.99	18.88 ± 14.69
Deficiency (<30 ng/ml)	65 (77.4%)	7 (70.0%)	58 (78.4%)
RBC folate (ng/ml)	424.26 ± 358.15	343.91 ± 467.89	436.95 ± 343.92
Deficiency (<140 ng/ml)	11 (25%)	3 (50%)	8 (21.1%)
Glucose-to-insulin ratio^∗^	37.21 ± 60.56	33.91 ± 45.17	37.67 ± 62.67

Data presented as the mean ± SD. No significant difference was found between the two groups. ^∗^Glucose unit (mg/dl) and insulin unit (*μ*IU/L).

**Table 5:** Prediction of crude birth weight by different parameters.

Variables	*β*	S.E.	Wald	*P* value	Exp (*β*)
*Binary logistic regression: model 1*					
Constant	−1.567	3.754	0.174	0.676	4.791
Age (yrs)	0.042	0.079	0.287	0.592	0.959
Gestational age (wks)	0.182	0.146	1.561	0.212	0.833
Parity	0.077	0.229	0.113	0.737	0.926
BMI	0.149	0.096	2.402	0.121	0.862
RBS	−0.003	0.013	0.068	0.795	1.003
FBS	−0.030	0.029	1.093	0.296	1.031
Triglyceride	−0.006	0.011	0.355	0.551	1.006
Vitamin B12	0.001	0.002	0.403	0.525	0.999
Vitamin D	0.003	0.024	0.015	0.902	0.997
*Binary logistic regression: model 2*					
Constant	−1.931	2.034	0.901	0.342	0.689
Age (yrs)	0.073	0.062	1.403	0.236	0.929
Gestational age (wks)	0.147	0.129	1.303	0.254	0.863
Vitamin B12	0.001	0.002	0.458	0.498	0.999
Vitamin D	0.008	0.024	0.114	0.736	0.992
*Binary logistic regression: model 3*					
Constant	1.285	0.592	4.709	0.030	0.277
Vitamin B12	0.001	0.002	0.572	0.449	0.999
Vitamin D	0.006	0.024	0.067	0.796	0.994

Birth weight category (LBW and NBW) considered as dependent variable. NBW considered as reference group. *P* value < 0.05 is considered statistically significant.

## Additional Points

*Highlights*. (i) Maternal nutrition in early pregnancy on metabolic status in newborns. (ii) Maternal underweight and metabolic status in pregnant mothers in early pregnancy.

## References

[1] Le Clair C., Abbi T., Sandhu H., Tappia P. S. (2009). Impact of maternal undernutrition on diabetes and cardiovascular disease risk in adult offspring. *Canadian Journal of Physiology and Pharmacology*.

[2] Morrison K. M., Anand S. S., Yusuf S. (2013). Maternal and pregnancy related predictors of cardiometabolic traits in newborns. *PLoS One*.

[3] Friis C. M., Qvigstad E., Roland M. C. P. (2013). Newborn body fat: associations with maternal metabolic state and placental size. *PLoS One*.

[4] Bazaes R. A., Salazar T. E., Pittaluga E. (2003). Glucose and lipid metabolism in small for gestational age infants at 48 hours of age. *Pediatrics*.

[5] Jaquet D., Trégouët D. A., Godefroy T. (2002). Combined effects of genetic and environmental factors on insulin resistance associated with reduced fetal growth. *Diabetes*.

[6] Barker D. J., Osmond C. (1988). Low birth weight and hypertension. *BMJ*.

[7] Barker D. J. P., Osmond C., Winter P. D., Margetts B., Simmonds S. J. (1989). Weight in infancy and death from ischaemic heart disease. *The Lancet*.

[8] Hales C. N., Barker D. J., Clark P. M. (1991). Fetal and infant growth and impaired glucose tolerance at age 64. *BMJ*.

[9] Simental-Mendía L. E., Castañeda-Chacón A., Rodríguez-Morán M., Guerrero-Romero F. (2012). Birth-weight, insulin levels, and HOMA-IR in newborns at term. *BMC Pediatrics*.

[10] McCance D. R., Pettitt D. J., Hanson R. L., Jacobsson L. T. H., Knowler W. C., Bennett P. H. (1994). Birth weight and non-insulin dependent diabetes: thrifty genotype, thrifty phenotype, or surviving small baby genotype?. *BMJ*.

[11] Dabelea D., Hanson R. L., Lindsay R. S. (2000). Intrauterine exposure to diabetes conveys risks for type 2 diabetes and obesity: a study of discordant sibships. *Diabetes*.

[12] Lindsay R. S., Nelson S. M., Walker J. D. (2010). Programming of adiposity in offspring of mothers with type 1 diabetes at age 7 years. *Diabetes Care*.

[13] Gluckman P. D., Hanson M. A., Pinal C. (2005). The developmental origins of adult disease. *Maternal & Child Nutrition*.

[14] Catalano P. M., Hauguel-De Mouzon S. (2011). Is it time to revisit the Pedersen hypothesis in the face of the obesity epidemic?. *American Journal of Obstetrics & Gynecology*.

[15] McKeigue P. M., Pierpoint T., Ferrie J. E., Marmot M. G. (1992). Relationship of glucose intolerance and hyperinsulinaemia to body fat pattern in south Asians and Europeans. *Diabetologia*.

[16] Ramachandran A., Snehalatha C., Vijay V. (2004). Low risk threshold for acquired diabetogenic factors in Asian Indians. *Diabetes Research and Clinical Practice*.

[17] Ehtisham S., Crabtree N., Clark P., Shaw N., Barrett T. (2005). Ethnic differences in insulin resistance and body composition in United Kingdom adolescents. *The Journal of Clinical Endocrinology & Metabolism*.

[18] Yajnik C. S., Lubree H. G., Rege S. S. (2002). Adiposity and hyperinsulinemia in Indians are present at birth. *The Journal of Clinical Endocrinology & Metabolism*.

[19] Gueri M., Jutsum P., Sorhaindo B. (1982). Anthropometric assessment of nutritional status in pregnant women: a reference table of weight-for-height by week of pregnancy. *The American Journal of Clinical Nutrition*.

[20] International Association of Diabetes and Pregnancy Study Groups Consensus Panel, Metzger B. E., Gabbe S. G. (2010). International association of diabetes and pregnancy study groups recommendations on the diagnosis and classification of hyperglycemia in pregnancy. *Diabetes Care*.

[21] Whitton C., Ho J., Tay Z. (2017). Relative validity and reproducibility of a food frequency questionnaire for assessing dietary intakes in a multi-ethnic Asian population using 24-h dietary recalls and biomarkers. *Nutrients*.

[22] Ramírez-Vélez R., López-Cifuentes M. F., Correa-Bautista J. E. (2016). Triceps and subscapular skinfold thickness percentiles and cut-offs for overweight and obesity in a population-based sample of schoolchildren and adolescents in Bogota, Colombia. *Nutrients*.

[23] Cornblath M., Hawdon J. M., Williams A. F. (2000). Controversies regarding definition of neonatal hypoglycemia: suggested operational thresholds. *Pediatrics*.

[24] Economides D. L., Proudler A., Nicolaides K. H. (1989). Plasma insulin in appropriate- and small-for-gestational-age fetuses. *American Journal of Obstetrics and Gynecology*.

[25] Preziosi P., Prual A., Galan P., Daouda H., Boureima H., Hercberg S. (1997). Effect of iron supplementation on the iron status of pregnant women: consequences for newborns. *The American Journal of Clinical Nutrition*.

[26] Yajnik C. S., Deshpande S. S., Jackson A. A. (2008). Vitamin B_12_ and folate concentrations during pregnancy and insulin resistance in the offspring: the Pune maternal nutrition study. *Diabetologia*.

[27] Pathak P., Kapil U., Yajnik C. S., Kapoor S. K., Dwivedi S. N., Singh R. (2007). Iron, folate, and vitamin B_12_ stores among pregnant women in a rural area of Haryana State, India. *Food and Nutrition Bulletin*.

[28] Carmel R., Mallidi P. V., Vinarskiy S., Brar S., Frouhar Z. (2002). Hyperhomocysteinemia and cobalamin deficiency in young Asian Indians in the United States. *American Journal of Hematology*.

[29] Yajnik C., Deshpande S. S., Lubree H. G. (2006). Vitamin B12 deficiency and hyperhomocysteinemia in rural and urban Indians. *The Journal of the Association of Physicians of India*.

[30] Xiao S., Hansen D. K., Horsley E. T. M. (2005). Maternal folate deficiency results in selective upregulation of folate receptors and heterogeneous nuclear ribonucleoprotein-E1 associated with multiple subtle aberrations in fetal tissues. *Birth Defects Research Part A: Clinical and Molecular Teratology*.

[31] Craciunescu C. N., Brown E. C., Mar M. H., Albright C. D., Nadeau M. R., Zeisel S. H. (2004). Folic acid deficiency during late gestation decreases progenitor cell proliferation and increases apoptosis in fetal mouse brain. *The Journal of Nutrition*.

[32] Hossain N., Khanani R., Hussain-Kanani F., Shah T., Arif S., Pal L. (2011). High prevalence of vitamin D deficiency in Pakistani mothers and their newborns. *International Journal of Gynecology & Obstetrics*.

[33] Godfrey K. M., Barker D. J. P. (2000). Fetal nutrition and adult disease. *The American Journal of Clinical Nutrition*.

[34] Lawlor D. A., West J., Fairley L. (2014). Pregnancy glycaemia and cord-blood levels of insulin and leptin in Pakistani and white British mother–offspring pairs: findings from a prospective pregnancy cohort. *Diabetologia*.

[35] Vrijkotte T. G. M., Algera S. J., Brouwer I. A., van Eijsden M., Twickler M. B. (2011). Maternal triglyceride levels during early pregnancy are associated with birth weight and postnatal growth. *The Journal of Pediatrics*.

